# Adsorption of methylene blue from textile industrial wastewater using activated carbon developed from *Rumex* *abyssinicus* plant

**DOI:** 10.1038/s41598-023-32341-w

**Published:** 2023-04-03

**Authors:** Jemal Fito, Mikiyas Abewaa, Ashagrie Mengistu, Kenatu Angassa, Abera Demeke Ambaye, Welldone Moyo, Thabo Nkambule

**Affiliations:** 1 Institute for Nanotechnology and Water Sustainability (iNanoWS), College of Science, Engineering, and Technology, University of South Africa, Florida Science Campus, Johannesburg, 1710 South Africa; 2 Department of Chemical Engineering, College of Engineering and Technology, Wachemo University, Hosanna, Ethiopia; 3 Leather and Leather Products Industry Research and Development Center, Addis Ababa, Ethiopia; 4 Department of Environmental Engineering, Addis Ababa Science and Technology University, Addis Ababa, Ethiopia

**Keywords:** Environmental sciences, Astronomy and planetary science, Engineering, Materials science

## Abstract

Methylene blue (MB) is abundantly found in textile industrial effluent which can cause severe health problems for public and environmental ecology. Therefore, this study aimed to remove MB from textile wastewater using the activated carbon developed from *Rumex* *abyssinicus*. The adsorbent was activated using chemical and thermal methods, and then it was characterized by SEM, FTIR, BET, XRD, and pH zero-point charge (pHpzc). The adsorption isotherm and kinetics were also investigated. The experimental design was composed of four factors at three levels (pH (3, 6, and 9), initial MB concentration (100, 150, and 200 mg/L), adsorbent dosage (20, 40, and 60 mg/100 mL), and contact time (20, 40, and 60 min)). The adsorption interaction was evaluated using response surface methodology. The characterization of a *Rumex* *abyssinicus* activated carbon was found to have multiple functional groups (FTIR), an amorphous structure (XRD), crack with ups and down morphology (SEM), pHpzc of 5.03 and a high BET-specific surface area of 2522 m^2^/g. The optimization of MB dye removal was carried out using the Response Surface methodology coupled with the Box Behnken approach. The maximum removal efficiency of 99.9% was recorded at optimum conditions of pH 9, MB concentration of 100 mg/L, the adsorbent dosage of 60 mg/100 mL, and contact time of 60 min. Among the three adsorption isotherm models, the Freundlich isotherm model was the best fit with an experimental value at R^2^ 0.99 showing the adsorption process was heterogeneous and multilayer whereas the kinetics study revealed that pseudo-second-order at R^2^ 0.88. Finally, this adsorption process is quite promising to be used at an industrial level*.*

## Introduction

Many textile industries are discharging huge volumes of wastewater into the nearby environment without proper treatment^1^. In the textile industry, many processes such as dyeing, finishing, and washing required a lot of water which makes the industry a water-intensive factory^2^. The textile industry is known to consume 1000 of the 100,000 types of dyes present in the commercial market. The annual production rate of dyes is estimated to be about 700,000 tons^3^. It was also reported that about 700,000 to 800,000 tons of dye with 100,000 distinct compounds are manufactured annually worldwide^4^. However, about 15% of the dyes used in industry are eventually released into the environment after being produced and processed^5^. Methyl orange, Rhodamine B, methylene blue (MB), Congo red, and Reactive Black-5 are classified into anionic, neutral, and cationic dyes which are among the most widely used dyes in the textile sector^6,7^. Dyes are coloring and valuable compounds for industrial products, particularly in textile industries to dye textiles, yarns, plastics, and other substrates. However, they are non-degradable due to chemical intricacy and multiplicity of smearing which results in distracting the environmental system^8^. Specifically, MB is a synthetic, heterocyclic aromatic, C_16_H_18_N_3_SCl 319.85 g/mol, (3,7-bis(dimethylamino) phenothiazine chloride tetra methylthionine chloride), and cationic chemical compound^9^. A large quantity of MB is used as a colorant for wool, silk, papers, cosmetics, temporary hair colorants, cotton, textile, food, and pharmaceutical industries^10^. MB is known for its antioxidant, cardio-protective, antimalarial, and antidepressant properties. Precisely, MB is a popular cationic dye and environmentally persistent, toxic, carcinogenic, and mutagenic chemical^10^. Introducing colored wastewater into the ecosystem is a notable cause of eutrophication, aesthetic pollution, and disruptions to aquatic life^11^. The wastewater generated from textile industries comprises several dyes and capable of causing serious health and environmental problems^12^. Thermal and photo stability of dye in the environment which results in absorption and reflection of sunlight. This reduces photosynthesis process and interferences with the natural flow of food chain. Long-term exposure to MB can cause significant health impacts such as anemia, cancer, vomiting, eye irritation, nausea, vomiting, methemoglobinemia, and mental confusion^13–19^. Therefore, the inevitable impact of these pollutants necessitates treatment prior to discharge to the mainstream and causing environmental degradation^20^.

The clean-up of the dyes from industrial wastewater was thoroughly studied through conventional wastewater. The commonly known conventional wastewater treatment technologies are composed of preliminary, primary, secondary, and tertiary treatment stages. These conventional wastewater treatment methods are inefficient to remediate MB from industrial effluents. The reason for low treatment performance is attributed low degradability of dyes chemically and biologically^21^. Hence, advanced wastewater treatment techniques such as advanced oxidation process, reverse osmosis, chemical precipitation, nanofiltration, membrane separation, electrocoagulation, ion exchange, membrane separation, photocatalysis, and electrodialysis are attracting the attention of scholars to overcome the shortcomings of conventional wastewater treatment techniques^22^. These technologies are efficient and effective for MB from textile industrial wastewater. However, these technologies have certain limitations such as requiring high energy, chemical consumption, operational cost running, huge capital inputs, high capital investment, and well-skilled technologists. This showed the discrepancy between the water quality demanded and the state of the art of the treatment technologies. Among many advanced treatment methods, adsorption is the most widely used technology due to its low cost, easy design, and environmentally friendly^23–27^. Adsorption is a surface phenomenon in which adsorbate is attached to the surface of the adsorbent. In adsorption, the selection of precursor material and the development of the adsorbent are important tasks in adsorption effectiveness. Traditional adsorbents can be classified as conventional and non-conventional groups but the ideal adsorbents are abundantly available, easy to prepare, cost-effective, non-soluble, eco-friendly, non-toxic, simple to regenerate, and efficient^28^. Practically, it could not be acquired such an idea adsorbent for water and wastewater applications. However, promising adsorbents can be regenerated easily, are socially acceptable, and are reusable at a minimum cost and effort. However, the basic quality and suitability of the adsorbent can be evaluated in terms of surface area, porosity, multi-functional groups, chemical composition, stable structures, and surface shape^29^. In the adsorption industry, one of the big challenges is the selection of precursor materials and the production of sustainable adsorbents. In general, adsorption is a promising technology that can be inherited in many sectors shortly^30^. But, acquiring the ideal adsorbent that can serve as multipollutant remediation from water and wastewater is a big challenge so far. On the other hand, activated carbons are the most studied and applied adsorbents due to their high surface area, surface functional groups, unique textural and chemical properties, and universality^31^. The industrial application of activated carbon is the first choice and is widely used. Its production was estimated to be 2757 × 10^3^ tons (5.7 billion USD) in 2021^32^. Normally, activated carbon is flexible for modification to improve surface structure and chemistry. Among all adsorbents, commercial activated carbons are the most effective adsorbent but they are very expensive to be widely used in wastewater treatment. The major challenge of activated carbon to be used at the industry level is the high production cost which is attributed to the cost of precursor material, chemical utilized and energy consumed. Hence, scientific community is searching for local available materials and laboratory based prepared activated carbon which could be cheap, abundantly available, effective and require minimum pretreatment^33^.

Many locally prepared activated carbons have been produced and used for the removal of various pollutants from industrial wastewater. For instance, Khat stems^34^, parthenium hysterophorus^25^, banana peduncle, seaweeds, mushroom compost^35^, bagasse fly ash^36^, and bentonite^26^. However, the adsorption performances of those adsorbents were significantly varied. These variations are partially attributed to adsorption factors such as pH, contact time, adsorbent dosage, adsorbate concentration, nature of the adsorbate, the specific surface area of adsorbent, etc.^33^. The application of the activated carbon for removal of the MB from the textile industry has been studied thoroughly^27^. For instance, parthenium hysterophorus activated carbon was used to remove MB from textile wastewater, and maximum removal efficiency of 93.8% was reported. In Another study, 96.7% removal efficiency of MB from textile wastewater was obtained^37^. Under a similar investigation, an MB adsorption capacity of 212.8 mg/g was recorded^18^. Even though those studies are promising, upgrading the adsorption surface area, longer contact time, minimum adsorption capacity, and scaling up the technology at the industrial level are serious limitations. Therefore, researchers are still searching for suitable precursor material to develop an ideal adsorbent. In line with this, *Rumex* *Abyssinicus* was proposed as promising biomass to produce an effective and efficient adsorbent to clean up MB from textile industrial effluent. Normally, Rumex Abyssinicus is a perennial herb, up to 3–4 m tall^38^. This plant is widely distributed in the highlands of tropical Africa, throughout North Africa, and Ethiopia^39^. So far various studies have been conducted to valorise *Rumex* *Abyssinicus*. For instance,^40^ evaluated the anti-inflammatory and antimicrobial nature of *Rumex* *Abyssinicus* and reported positive findings. On the other hand, the application of *Rumex* *Abyssinicus* in tannery processing was investigated by^41^, its application in the preservation of goat skin^42^, and the chemical composition of *Rumex* *Abyssinicus* plant which later applied for antifungal, antibacterial, and antioxidant^43^ are among studies conducted on utilization of various parts of *Rumex* *abysiniccus*. However, the plant has never been investigated as adsorbent for wastewater treatment. It is supposed that Rumex Abyssinicus emerged with a huge adsorption potential to develop the ideal adsorbent for removal of MB from textile wastewater. Therefore, this study aimed at investigating the performance of activated carbon produced from *Rumex* *Abyssinicus* for adsorption of MB dye from textile industrial wastewater using response surface methodology coupled with box Behnken design by four factors at three levels. The experimental factors with corresponding levels are pH (3, 6, and 9), contact time (20, 40, and 60 min), initial MB concentration (100, 150 and 200 mg/L), and adsorbent dosage (20, 40, and 60 mg/100 mL). The full factorial experimental design of 3^4^ was used but the Box Behnken approach was implemented to reduce the number of experiments to 30. Finally, interaction effects among various variables were determined using the response surface methodology.

## Materials and methods

### Adsorbent preparation

**Figure 1:**
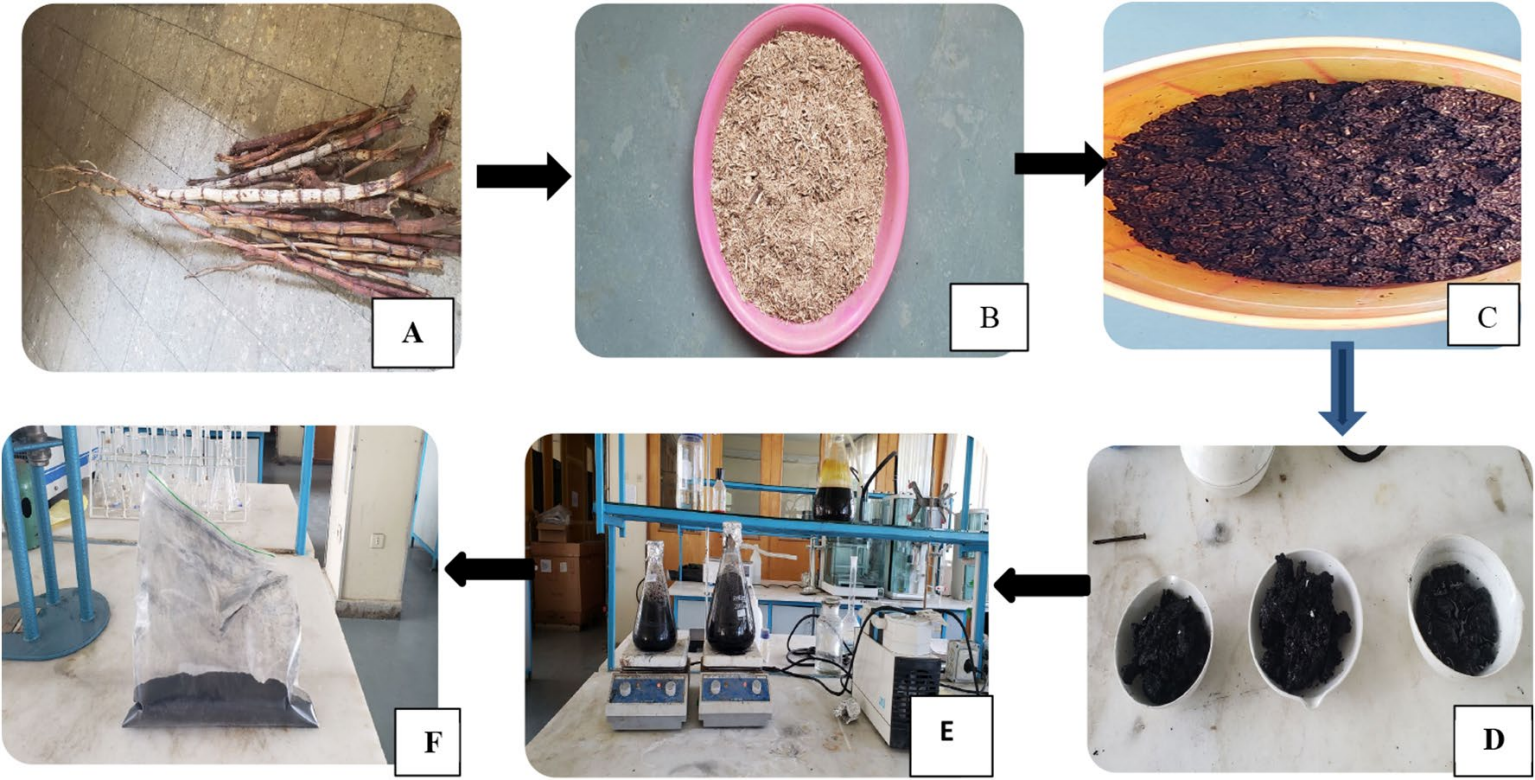
HYPERLINK "sps:id::fig1||locator::gr1||MediaObject::0" Different preparation stages of activated carbon from *Rumex* *abyssinicus.*

*Rumex* *Abyssinicus* was collected from Addis Ababa Science and Technology University, Addis Ababa, Ethiopia. The geographical location and metrological condition of Addis Ababa Science and Technology are described by high-altitude of 2300 m (8°58′ N and 38°47′ E) above sea level with an annual mean temperature of 15.9 °C, rainfall of 1089 mm and relative humidity of 60.7%. The voucher of the plant specimen (with no ID) was deposited in a university herbarium (Addis Ababa University) and the plant specimen was collected by the researcher (Mikiyas Abawaa) and was crossed checked against the herbarium. The plant identification was performed by an expert assigned to the herbarium site. The collected *Rumex* *abyssinicus* was cut into small pieces of 5–10 mm size using a knife before being washed several times with distilled water. The washing of the collected sample was intended to remove any debris that may have been adhered to the surface of the material. After washing, the sample pieces were dried using the oven at 105 °C for 24 h. The dried samples of *Rumex* *abysinicus* were impregnated with concentrated phosphoric acid (88%) in the ratio of 1:3 by weight and soaked for 24 h at room temperature. Afterward, the impregnated sample was allowed to dry in an oven at 105 °C for 24 h. Then, the dried impregnated sample was pyrolyzed in a muffle furnace at 600 °C for 2 h. The pyrolyzed sample was allowed to cool in the desiccator and washed several times with distilled until the pH of the adsorbent becomes nearly neutral. The pH-adjusted activated sample was oven dried, ground into 250 µm, and stored in an airtight plastic container until utilized for the adsorption process^22^. Figure 1 depicts different *Rumex* *abyssinicus* activated carbon preparation stages such as raw *Rumex* *abyssinicus* (A), reduced *Rumex* *abyssinicus* (B), phosphoric acid impregnated *Rumex* *abyssinicus* (C), pyrolyzed *Rumex* *abyssinicus* (D), washing stage of *Rumex* *abyssinicus* to remove H_3_PO_4_ (E) and ready-made activated carbon (F).

### Adsorbent characterization

#### Proximate analysis

*Proximate* *analysis* of certain activated refers to moisture content, ash content, volatile matter, and fixed carbon of the material. American Standard for testing materials was used for proximate analysis of the prepared activated carbon. For moisture content determination, 1 g of *Rumex* *Abyssinicus* activated carbon was added to the crucible. Then, the sample was placed in the oven at 105 °C for 24 h. Afterward, the sample was allowed to cool in a desiccator at room temperature. Then, the difference between the initial and final weight was used to calculate the moisture content. Equation (1) was used to calculate the moisture content of the adsorbent^44^.

1 $$\mathrm{MC}=\frac{{\mathrm{W}}_{1}- {\mathrm{W}}_{2} }{{\mathrm{W}}_{1}} \,  \times \, 100 {\%}$$

where W_1_ refers to the weight of the crucible plus activated before drying, W_2_ indicates the weight of the crucible plus activated after drying and MC is the moisture content of the adsorbent

On the other hand, for volatile matter determination, 1 g of the adsorbent was added to the crucible after which it was ignited in the muffle furnace at 800 °C for 8 min. Equation (2) was used to compute the volatile matter content of the *Rumex* *abyssinicus* adsorbent^34^**.**

2 $$\mathrm{VM }= \frac{ {\mathrm{W}}_{1}- {\mathrm{W}}_{2} }{{\mathrm{W}}_{1}} \, \times \, 100{\%}$$

where VM is the volatile matter content, W_1_ is the weight of the crucible plus adsorbent before ignition, and W_2_ is the weight of the crucible plus adsorbent after ignition. Similarly, the ash content of prepared activated carbon was determined by the ignition method. In this process, 1 g of *Rumex* *abyssinicus* activated carbon was placed in a crucible which was then ignited in a muffle furnace at 550 °C for 4 h. Then, the sample was placed in the desiccator to avoid moisture absorption and contamination. The ash content of the adsorbent was determined using Eq. (3)^44^.

3 $$\mathrm{AC}= \frac{{\mathrm{W}}_{2}}{{\mathrm{W}}_{1}} \times 100{\%}$$

where AC is the ash content, W_2_ is the weight after ignition, and W1 is the weight before ignition. Finally, fixed carbon content was determined using Eq. (4)^34^.

4 $$\mathrm{FC} ({\%})=100-(\mathrm{MC}+\mathrm{VM}+\mathrm{AC}) $$

where FC is the fixed carbon, MC is the moisture content, VM is the volatile matter and AC is the ash content

#### pH points of zero charge

The pH of the adsorbent equals its pH point of zero charges, and the surface charge of the adsorbent becomes neutral. This phenomenon is called the pH point of zero charges. On the other hand, there will be an equal number of positively and negatively charged surfaces. The mass titration method was used for the determination of the pH point of a zero charge of *Rumex* *abyssinicus*-derived activated carbon. In this process, 0.1 g of adsorbent was added to several flasks containing 50 mL of 0.5 M NaCl solution. The pH of these samples was adjusted to 2, 4, 6, 8, 10, and 12 using 0.1 M NaOH or 0.1 M HCl. Then, the samples were shaken using an orbital shaker for 24 h, after which the pH final of each solution was determined. Finally, the pH point of zero charges of the material was determined by drawing the graph of pH initial versus pH final. The point at which pH final and pH initial overlapped was recorded as the pH point of zero charges^26^.

#### Scanning electron microscope (SEM)

SEM was used for the examination of the surface morphology of the adsorbent. The regular machine’s operating protocol was followed for sample preparation and scanning. The machine used in the current study was the JCM-6000PLUS Benchtop SEM (JOEL), Japan. For the scanning of the surface morphology of the adsorbent, a 20 ${\upmu {\rm m}}$ resolution and 10 kV energy were used. The sample was scanned by placing it on the carbon tape and running the machine at an 8-mm working distance. The SEM was operated at 10 A current, 10 kV operational energy, and a 1500 times magnification^24,45^.

#### Fourier transform infrared (FTIR)

FTIR was used to determine functional groups present in the prepared adsorbent. The KBr method was used for FTIR analysis of the adsorbent. In this process, the prepared adsorbent was thoroughly mixed with KBr with a ratio of 2:200. Then, the homogenous sample of the mixture was obtained by crushing in the mortal. Afterward, a very fine plate was produced using the molder. The FTIR spectrophotometer (FTIR, Thermo Nicolet 5700, and Waltham, MA, USA) was subsequently used to perform the plate analysis. A wavelength range of 4000–400 cm^−1^ was used for the FTIR analysis^24,35^.

#### Brunauer–Emmett–Teller (BET)

BET was used to calculate the prepared adsorbent's specific surface area. In this procedure, the three sample preparation tubes each containing 0.4 g of the adsorbent were used. Horiba's SA-9600 Series and surface area analysis machine were utilized to calculate the adsorbent's precise surface area. For this investigation, the machine was run for 1 h at a degassing temperature of 200 °C. Using a surface area analyzer and the isotherms of nitrogen gas adsorption and desorption at 700 mm atmospheric pressure, the surface area was estimated. To improve N_2_ adsorption on the surface of the adsorbent, liquid nitrogen at −196.5 °C was used. The adsorbent's BET-specific surface area was then calculated using the p/p0 ratio^26^.

#### X-ray diffraction (XRD)

XRD was used to determine the crystalline nature of the prepared adsorbent. This technique is a powerful non-destructive technique that characterizes the presence of crystalline materials with a diffraction angle of 2θ from 2° to 80° using the OLYMPUS BTXH X-ray diffraction instrument. The analysis was performed under the following conditions: initialization power of 15 kV, 5 mA, and a fixed wavelength of 1.541 nm. Finally, the crystalline structure of the sample was determined based on the observed peaks^46^.

### Batch adsorption experiment

The three working solutions (100, 125, 150 mg/L) of MB were prepared by dissolving 100 mg, 125 mg, and 150 mg of MB dye in 1000 mL of distilled water respectively. In the preliminary study, experimental design and MB concentration of real textile wastewater was used to fix at the above-mentioned concentrations. In adjusting the solution pH, 0.1 M NaOH and 0.1 M HCl were used throughout the experimental study. The experimental design of this study is based on the four independent factors (initial MB concentration, pH, adsorbent dosage, and contact time) at three levels as indicated in Table 1. The lower level was assigned as −1, whereas the middle and higher levels are represented by 0 and + 1 respectively. Combining the lower, middle, and upper values the full factorial experimental design of 3^4^ will result in 81 experimental runs. However, the Box Behnken approach of response surface methodology experimental design was used to fix the number of experiments to 30. Triplicate sample analyses were performed and the average value was reported. Box Behnken's approach of response surface methodology not only minimizes the costs but also reduces the time to be spent with better experimental results^47^. The selection of treatment conditions was undertaken randomly to avoid experimental bias. The batch adsorption experiment was undertaken by agitating the adsorption solution at a fixed contact time. The concentration of MB was determined using a UV–Visible spectrophotometer at a wavelength of 668 nm. Finally, the removal efficiency of MB and the adsorption capacity of the adsorbent were determined using Eqs. (5) and (6) respectively^27,44^.

5 $$\mathrm{R}\left({\%}\right)=\left(\frac{{\mathrm{C}}_{\rm{i}}-{\mathrm{C}}_{\rm{f}}}{{\mathrm{C}}_{\rm{i}}}\right) \times 100$$

6 $$\mathrm{Qe}=\left(\frac{\mathrm{Co}-\mathrm{Ce}}{\mathrm{m}}\right) \times \mathrm{m}$$

where %R is the MB removal percentage, Q_e_ is the amount of MB adsorbed per unit mass of the adsorbent (mg/g), Co is the initial MB concentration (mg/L), C_f_ is the final MB concentration (mg/L), Ce is the concentration of MB at equilibrium, V is the volume of the aqueous solution (mL) and M is the dry mass (g)

**Table 1:** Full factorial experimental design with factors and levels.

Variables	Low (−)	Middle (0)	High (+ 1)
pH	3	6	9
Adsorbent dose (mg/100 mL)	20	40	60
MB concentration (mg/L)	100	150	200
Contact time (min)	20	40	60

### Adsorption isotherms

Adsorption isotherms are equilibrium relationships describing how pollutants interact with the adsorbent materials. This phenomenon is an important condition that expresses the surface properties and capacities of the adsorbent. Adsorption isotherms are used for the optimization of the adsorption mechanism and for effectively designing the adsorption systems. There are various adsorption isotherm models which are widely used to determine the relationship between adsorbent and adsorbate at equilibrium. Langmuir, Freundlich, Dubinin–Radushkevich, Temkin, and Toth are among the widely used adsorption isotherm models. In the current study, the equilibrium adsorption isotherm models were evaluated at constant optimum conditions of pH 9, contact time 60 min, adsorbent dose 60 mg/L, and varying the initial MB concentrations from 100 to 200 mg/L i.e. (100, 120, 140, 160, 180 and 200)^48,49^. The Langmuir isotherm model assumes monolayer and homogenous surface adsorption. Equation (7) presents the general equation of the Langmuir adsorption isotherm model, where the linearized form is shown in Eq. (8)^22^.

7 $$\mathrm{qe}=\frac{{\mathrm{K}}_{\rm{L}}{\mathrm{q}}_{\rm{max}}{\mathrm{C}}_{\rm{e}}}{1+{K}_{L}\mathrm{Ce}}$$

8 $$\frac{1}{\mathrm{qe}}= \frac{1}{{\mathrm{q}}_{\rm{max}}}+\frac{1}{{\mathrm{K}}_{\rm{L}}{\mathrm{q}}_{\rm{max}}{\mathrm{C}}_{\rm{e}}}$$

In Eq. (7), K_L_ (L/mg) refers to the Langmuir constant related to the free energy of adsorption, whereas, ${q}_{max}$ (mg/g) is a maximum monolayer adsorption capacity of the adsorbent. Furthermore, Eq. (9) depicts the dimensionless separation factor constant (RL)^50^. This factor is used to estimate Langmuir's isothermal feasibility.

9 $$\mathrm{RL}=\frac{1}{1+{\mathrm{K}}_{\rm{L}}\mathrm{Ce}}$$

One of the widely used adsorption isotherm models is the Freundlich isotherm model which assumes the adsorption process takes place on a heterogeneous surface. On the other hand, in the Freundlich adsorption isotherm model, the nature of the adsorption process is multilayer. Equation (10) is the general equation for the Freundlich isotherm model, whereas the linearized form is presented in Eq. (11)^46^.

10 $${\mathrm{q}}_{\rm{e}}={\mathrm{K}}_{\rm{F}}{\mathrm{Ce}}^{\frac{1}{\mathrm{n}}}$$

11 $$\mathrm{logqe}={\mathrm{logK}}_{\rm{F}}+1/\mathrm{n logCe}$$

K_F_ denotes adsorption capacity (mg/g), and 1/n denotes an empirical parameter related to adsorption intensity, with a value between 0 and 1 denoting favorable conditions. Moreover, a value of 1/n indicates the adsorption process to be cooperative if > 1, independent of concentration if = 1, and normal if < 1. As per the Temkin isotherm model, the surface coverage resulting from the interaction of adsorbent and adsorbent will result in the linear decrease of the heat of adsorption. The Temkin isotherm is presented by (12)^44^.

12 $${\mathrm{q}}_{\rm{e}}=\frac{\mathrm{RT}}{\mathrm{BT}}\mathrm{lnAT}+\frac{\mathrm{RT}}{\mathrm{BT}}\mathrm{lnCe}$$

The value of BT (Temkin constant i, e heat of adsorption), AT equilibrium binding constant, R is the universal gas constant and T is the temperature of the system. AT and BT are computed from a plot of qe vs $\mathrm{lnCe}$.

### Adsorption kinetics

The rate at which a solute is retained or released from an aqueous environment to a solid-phase interface for a particular adsorbent dose, temperature, flow rate, and pH is represented by an adsorption kinetics curve. Kinetics study is of great significance. It provides information like the solute uptake rate, which intern used to establish the residence time required for the completion of the adsorption process. Pseudo-first-order, Pseudo-second-order, and Intraparticle diffusion models are well-known adsorption kinetic models. In the current study, the adsorption kinetics was established at fixed pH of 9, the adsorbent dosage of 60 mg/100 mL, the initial MB concentration of 100 mg/L, and varying contact times at 10, 20, 30, 40, 50, and 60 min. The three kinetic models i.e. Pseudo-first-order, Pseudo-second-order, and Intraparticle diffusion are presented by Eqs. (13), (14), and (15) respectively^51^.

13 $$Log (qe-qt) = log (qe)- \frac{{K}_{1}t}{2.303}$$

The plot of *t* vs $\mathrm{log}(qe-qt)$ results in a slope of $-\frac{\mathrm{K}1\mathrm{t}}{2.303}$ and intercept of log (qe). Similarly in the pseudo second order kinetics, the slope becomes 1/qe and the intercept will be 1/k_2_qe^2^ as t/qt is plotted against t^51^.

14 $$\frac{t}{qt}=\left(\frac{1}{qe}\right)t+\frac{1}{{K}_{2}{{q}_{e}}^{2}}$$

In the Intraparticle diffusion model, Kp is determined from the intercept of log t against log qt^36^**.**

15 $$\mathrm{log}\left(qt\right)=\mathrm{log}\left(Kp\right)+0.5\mathrm{log}\left(t\right)$$

where *q*_*e*_ is the mass of MB adsorbed at equilibrium (mg/g), *q*_*t*_ is the mass of dye adsorbed at time t (mg/g), *K*_1_ is the Pseudo-first-order constant (min^−1^), *K*_2_ is the Pseudo-second-order constant (g/mg/min), and *K*_*p*_ the constant value (mg/g/min^0.5^).

### Ethical approval and consent to participate

All methods are carried out according to the institution’s guidelines and regulations. All experimental protocols were approved by the institution’s ethical clearance committee. Finally, all experimental studies on plants have complied with relevant institutional, national, and international guidelines and legislation. Experimental research and field studies on plants, including the collection of plant material complied with the institution’s guidelines and regulations. During sample collection, our institute gave a support letter to give permission for sample collection and the textile industry owners gave permission for sample collection and in situ measurements.

## Results and discussion

### Adsorbent characteristics

#### Proximate analysis

The moisture content, ash content, volatile matter, and fixed carbon of the prepared adsorbent were analyzed and the results of proximal values were reported in terms of mean plus standard deviation as indicated in Table 2. These values were determined to be moisture content 2.95, volatile matter 18.74, ash content 9.82, and fixed carbon 68.49%. These values are within a range of standard quality for proximate analysis of activated carbon. Normally, fixed carbon is the most essential parameter that determines the quality of the activated carbon. It is known that for activated carbon to be effective and efficient in adsorbing multipollutant from wastewater, the fixed carbon has to be as high as possible. This is because fixed carbon refers to the carbonous material that can play a significant role in adsorbing many pollutants. As indicated by the study conducted by^52^, the fixed carbon percentage of the prepared adsorbent should be at least 60%. Hence, the value of the current study is in agreement with the above scenario. However, the ash content of the adsorbent is an inert part of activated that can be composed of oxides mainly which are not contributing to the adsorption of pollutants. These oxides are not only simply increasing the mass of the adsorbent but also reduce the adsorption performance of the adsorbent by occupying the adsorbent active site where the pollutants are expected to be attached thereby decreasing the specific surface area of the adsorbent. Hence, higher fixed carbon is expected to be a promising phenomenon for adsorption. According to^52^ the maximum ash content set for activated carbon is 10%. This suggests that the current produced activated carbon is in good agreement with the high-quality standard. The currently prepared activated carbon is better in terms of its proximal values compared to many locally produced adsorbents^25,27,34^. Eventually, an activated carbon having less ash content, volatile matter, and moisture content with a high percentage of fixed carbon is a suitable material for adsorption technology.

**Table 2:** Proximate values for *Rumex* *abyssinicus* activated carbon.

Parameter	Values (%)
Moisture content	2.95 $\pm 0.61$
Ash content	9.82 $\pm 0.35$
Volatile matter	18.74 $\pm 1.12$
Fixed carbon	68.49 $\pm 1.54$

#### Point of zero charge analysis

**Figure 2:**
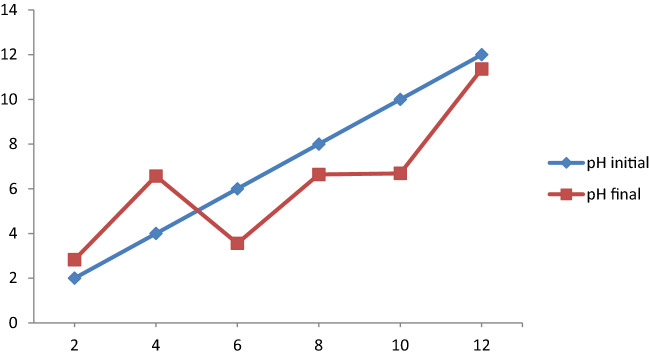
pHpzc of *Rumex* *abyssinicus* activated carbon.

The pHpzc of the prepared adsorbent was determined to be 5.03 as shown in Fig. 2. At a pH value of 5.03, the surface density of the adsorbent is zero. This can be defined in such a way that there is an equal number of positively and negatively charged surface charges. Moreover, at pH above 5.03, the surface of the adsorbent is negatively charged. On the other hand, at pH below 5.03, the surface of the adsorbent is positively charged. Normally adsorption of anions is favoured at the pH of a solution less than the pHpzc whereas that of cations is promising at the pH of a solution greater than pHpzc. Therefore, cationic dyes like MB are expected to be adsorbed sufficiently at the negatively charged surface density of the adsorbent. Hence, the removal MB is favoured at the pH of the solution above 5.06. Similar observations were reported for various activated carbons synthesized from different raw materials. For instance, the pHpzc of commercial AC powders (ACS25) of 5.0^53^, the pHpzc of oil palm trunk-derived activated carbon of 4.8^54^, the pHpzc for Leucaena leucocephala seed pod activated carbon of 5.20^55^, the pHpzc of granular activated carbon of 4.89^56^ and rice husk activated carbon-supported Zink oxide of 5.10 was reported^57^.

#### Surface morphology analysis

**Figure 3:**
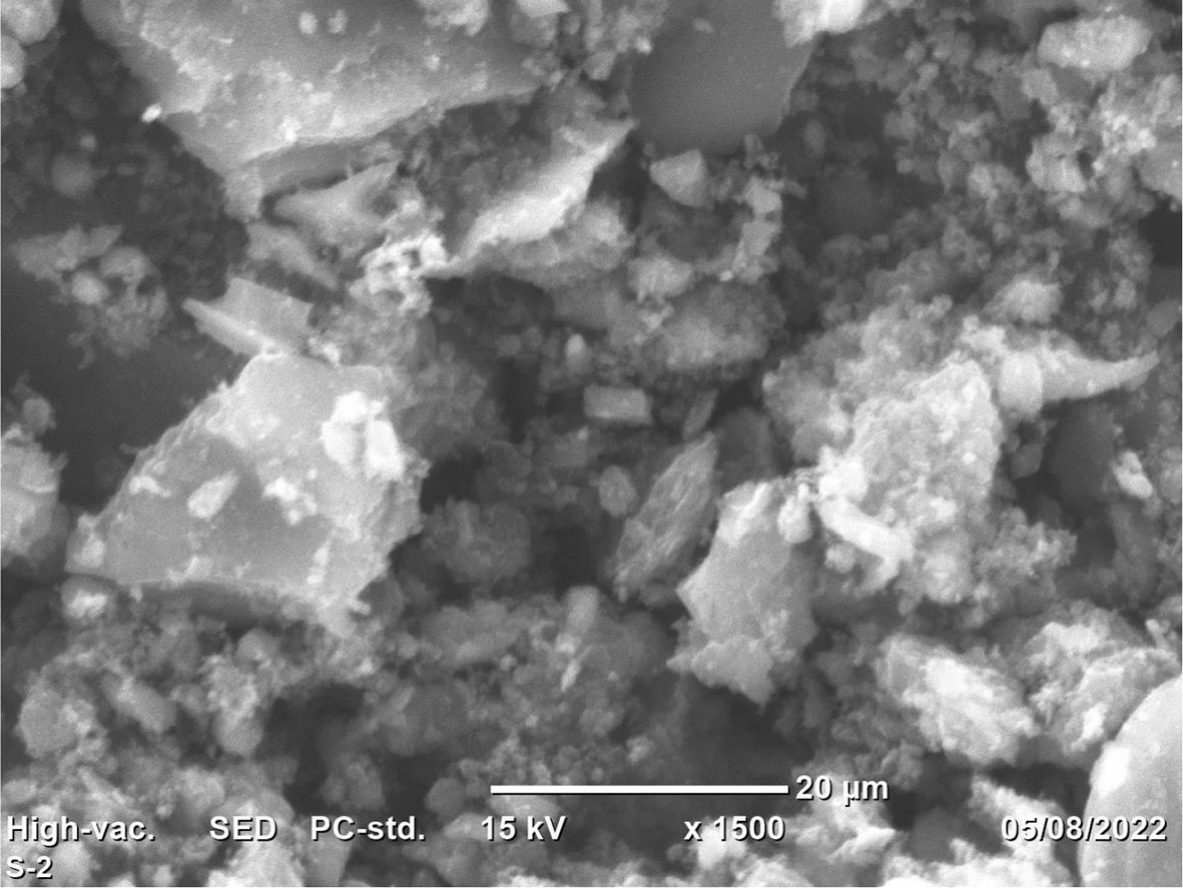
SEM morphology of *Rumex* *abyssinicus* adsorbent.

The surface morphology of the prepared activated carbon was evaluated using a Scanning Electron microscope and the finding of the study is presented in Fig. 3. The scanning of the surface of the adsorbent was conducted at a resolution of 20 $\mathrm{\mu m}$ and magnification of 1500 times. It was found that the adsorbent is highly porous, heterogeneous, and course surface with fluffy and rough microstructure. The morphology of the adsorbent is in good agreement with the adsorbent surface area. It is evident from the image that there are morphological cracks on the surface of the adsorbent. Moreover, the highly porous structure of the prepared adsorbent is the fertile precondition for the adsorption of MB dye. Previously conducted studies^24,25,27,34^ have reported similar findings.

#### FTIR analysis

**Figure 4:**
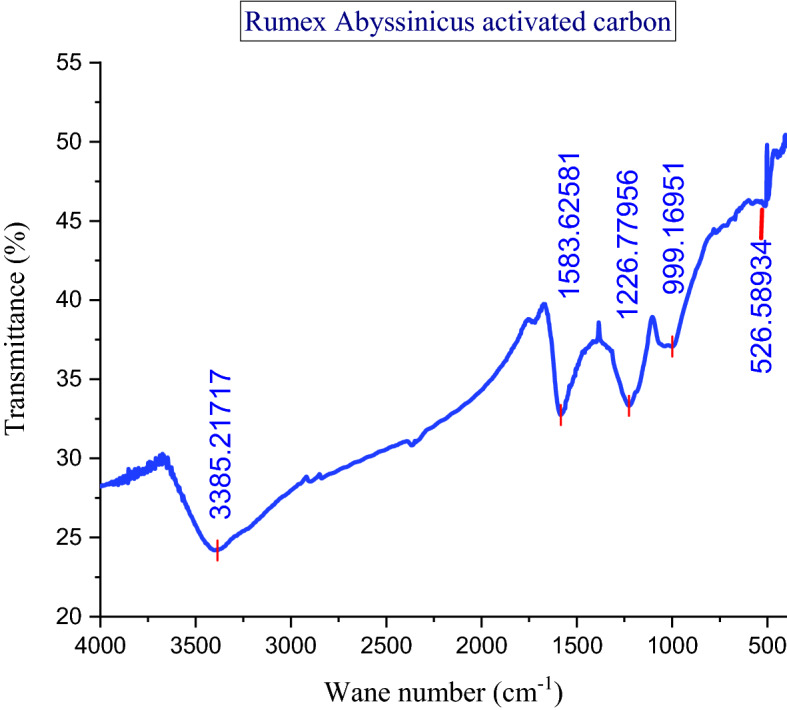
FTIR Analysis of *Rumex* *abyssinicus* activated carbon.

The prepared adsorbent was found to be composed of many functional groups capable of adsorbing MB from textile wastewater. It can be observed from Fig. 4 that five major peaks were found from the analysis of FTIR. These peaks are observed at the spectrum of 3385, 1583, 1226, 999, and 524 cm^−1^. Oxygen-containing functional groups such as carboxyl and hydroxyl create a conducive environment for the adsorption of MB. The stretching peak observed at 3385 cm^−1^ is attributed to O–H groups. The C=O bond spectrum is indicated in the peak observed at 1583 cm^−1^. Moreover, the peak observed at 1226 cm^−1^ would indicate the presence of carboxylic groups –COOH with its derivatives possibly such as carboxylates, the peak of 999 cm^−1^ would correspond to the C–O bond which is frequently associated with ether group −C−OC− and the C–OH found in celluloses. On the other hand, the peak at 524 cm^−1^ represents the alkene groups which are supposed to be with an insignificant contribution. In another study of activated carbon for surface functionalities, a similar finding was reported by Mohammed et al.^41,42^ and Kengne et al.^43^. Finally, the presence of multiple functional groups on the surface of this activated carbon is the fertile precondition for the effective adsorption of MB from industrial effluent.

#### Specific surface area analysis

The specific surface area of the adsorbent was analyzed using the BET and the result of the analysis was determined to be 2522 m^2^/g. This very high-specific surface area for activated carbon-prepared adsorbent makes the adsorbent a potential candidate to be used in the adsorption process. Normally, the presence of multiple functional groups coupled with a very porous structure and high specific surface area is the crucial component of high-quality adsorbents. Essentially, this activated carbon is the ideal adsorbent when evaluated in terms of the abundance of the precursor material, non-soluble, efficient, easy to prepare, and eco-friendly. However, adsorbent regeneration, cost-effectiveness, and compositing magnetic material for separation would be the next investigation. The other critical issues that need further improvement are economic efficiency and low energy consumption during adsorbent preparation and regeneration. Compared to many locally prepared activated carbons, the current activated carbon is superior in terms of the specific surface. For instance, the specific surface area of bentonite is 265 m^2^/g^26^, Parthenium hysterophorus-derived activated carbon is 268 m^2^/g^25^, Fe_3_O_4_-GO is 296 m^2^/g^24^ and sugarcane bagasse is 2236.93 m^2^/g^58^. However, the surface area of the recently emerged adsorbent metal–organic framework is extremely high in the range of 1000–10,000 m^2^/g with good properties such as superior performances, tunability structure, thermal stability, and mechanically dispersible^59–61^. The limitation of those materials is instability in an aqueous solution.

#### Crystalline structure analysis

**Figure 5:**
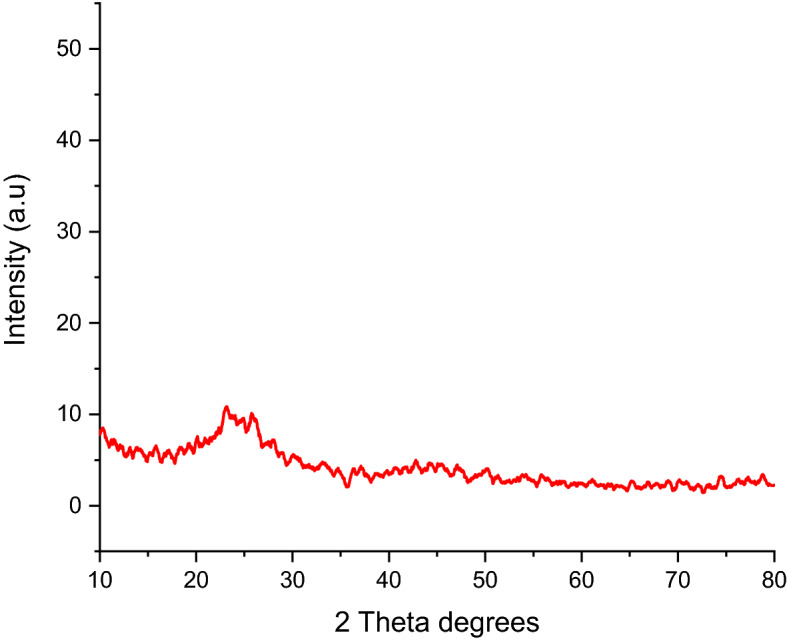
XRD image of *Rumex* *abyssinicus* activated carbon.

The crystalline nature of the adsorbent was examined using XRD. The *Rumex* *Abyssinicus*-derived activated carbon was found to have an amorphous structure as illustrated in Fig. 5. Normally, the thermal activation and chemical activation of pristine activated carbon would result in denaturing the crystalline nature of the material. Amorphous materials typically exhibit increased surface acidity and specific surface area. These characteristics strengthen the bond between the adsorbent and substrate material which often results in more adsorbed material. However, the interaction of adsorbate with crystalline structure is weak because of the significant impact that crystal structure has on the adsorption process^62^.

### Optimization of MB adsorption

The removal efficiency of MB dye is ranged from 82.2 to 99.9% as indicated in Table 3. The maximum removal efficiency of 99.9% was attained at the optimum conditions of pH 9, contact time of 60 min, the adsorbent dosage of 60 mg/100 mL, and initial MB concentration of 100 mg/L. However, the maximum adsorption capacity of 322 mg/g was recorded at a pH of 9, contact time of 60 min, adsorbent dose of 60 mg/100 mL, and MB concentration of 200 mg/L. Moreover, a small amount of adsorbent dosage (60 mg/100 mL) was utilized to remove 99.9% of the MB. This makes the prepared adsorbent effective even at a lower dosage. Shifting the initial MB concentration from the optimum value to the higher one, while keeping the other parameters at optimum, has resulted in the removal efficiency of 98.9%. This result has shown an increment of 0.97%. On the other hand, changing the adsorbent dosage from 60 to 20 mg/100 mL when the other three parameters are kept at optimum resulted in a removal efficiency of 98.8%. This accounts for a 1.15% reduction in removal efficiency. More importantly, this suggests that the effect of adsorbent dosage is higher compared to the initial MB concentration. Finally, the removal efficiency of MB increases with increasing adsorbent dosage, pH, and contact time. However, increasing the initial MB concentration decreases removal efficiency.

**Table 3:** Adsorption performance *Rumex* *abysiniccus* activated carbon for MB removal.

Run	pH	MB concentration (mg/L)	Adsorbent dosage (mg/100 mL)	Contact time (min)	Removal efficiency (%)
1	3	200	20	20	82.16
2	3	100	60	20	99.12
3	6	100	40	20	98.86
4	3	200	40	20	91.46
5	9	200	20	20	88.26
6	6	100	20	20	96.01
7	3	150	20	20	87.04
8	6	200	60	20	99.31
9	3	150	20	20	87.87
10	6	200	40	20	93.95
11	3	150	60	40	98.86
12	9	100	60	40	99.56
13	6	100	40	40	98.62
14	3	150	40	40	95.23
15	6	200	60	40	99.14
16	6	150	20	40	93.08
17	3	100	20	40	94.35
18	9	100	40	40	98.98
19	6	100	60	40	99.06
20	9	150	20	40	96.02
21	9	100	60	60	99.96
22	3	150	20	60	94.28
23	9	200	60	60	98.99
24	9	100	40	60	99.24
25	9	200	20	60	94.25
26	9	100	20	60	98.81
27	9	200	60	60	99.11
28	6	200	60	60	99.08
29	3	100	20	60	97.98
30	6	200	60	60	99.32

MB removal by the activated carbon was investigated by many researchers and different experiences was recorded. Many removals of MB were studied under aquatic solutions which are difficult to acquire the same results under the application of real wastewater. For instance, the stem of Buxus Wallichiana activated carbon the maximum adsorption capacity of 866.15 mg/g for MB removal from aqueous solution was reported^4^. In another study, a small amount of adsorption capacity of 11.4 for MB removal was recorded^27^. Basically, the comparison of the adsorption performance of the activated carbons should include the types of solution used either aqueous solution or real wastewater, the strength, common pollutants effect, surface area of the adsorbent, pollutant concentration, and experimental condition. Compared to many locally prepared activated carbons the currently prepared activated carbon shows superior performance. The adsorption studies on MB removal are supposed to be checked the performance of real wastewater with the dye. From this discussion, the surface area, and the performance of the current activated carbon, it can be possible to conclude the prepared ACs are the best choice to represent commercial activated carbon. However, further investigations of activated carbon are needed before pilot scale and commercialization purposes. Moreover, many more studies are still suffering from excessive use of adsorbents for the removal of small amounts of MB. Hence the current study serves as an alternative method of removing MB from wastewater using a small dosage of adsorbent. This makes the current study more economical. Table 4 presents the experimental results for the removal efficiency of MB from wastewater or aqueous solution.

**Table 4:** Comparison of MB removal efficiencies of different studies.

Adsorbents	%Removal efficiency/adsorption capacity mg/g	Source
Parthenium hysterophorus	94%	^27^
ZnO nanoparticles	72%	^63^
Periodiated modified nanocellulose	78%	^64^
Kaolin	98%	^65^
Graphene oxide aerogel	95%	^66^
Scrap tire	91%	^67^
Parthenium hysterophorus activated carbon	11.4	^27^
Scrap tire-derived activated carbon	73.5	^67^
Kaolin	1.9	^65^
Zeolite	1.7	^65^
Periodiated modified nanocellulose	90.9	^64^
Barely bran	63.2	^68^
Enset midrib leaf	35.5	^68^
*Rumex* *abyssinicus*-derived activated carbon	384/99.9%	Current study

### Interaction effects on the MB removal efficiencies

#### Initial MB concentration and adsorbent dosage

**Figure 6:**
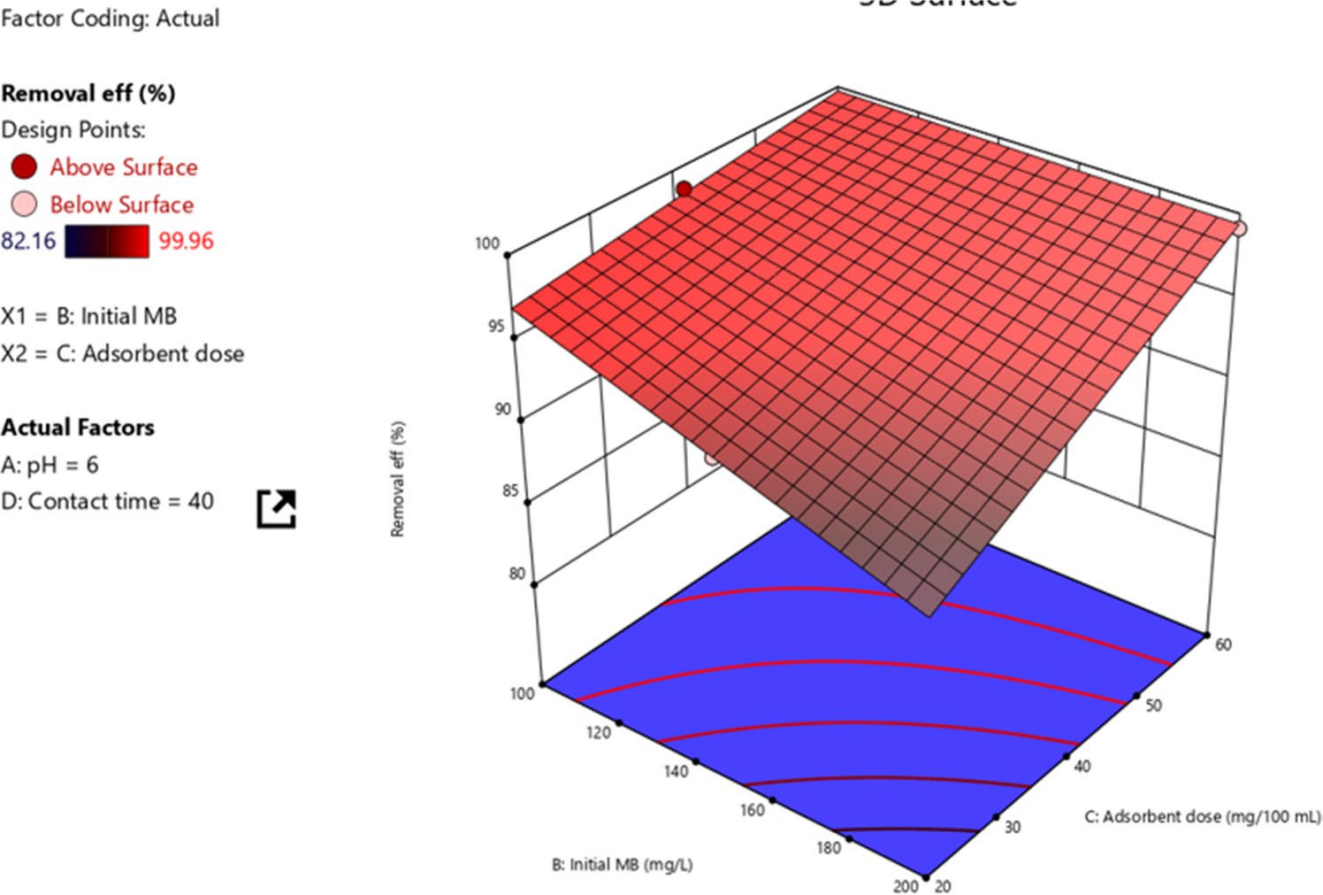
Interaction effects of initial MB concentration and adsorbent dosage.

The removal efficiency of pollutants from the wastewater or an aqueous solution not only depends on the individual effects but also the interaction effects of two or more variables. The statistical analysis suggested that the interactive regression model best fits the removal efficiency. Moreover, among the six interactions that exist between the four independent variables; five of them are found to be significant. The only insignificant interaction as per this model is the one between pH and initial MB concentration. As can be observed from Fig. 6 the interaction of initial MB concentration and adsorbent dosage was found to have a positive impact on the removal efficiency of MB. Individually increasing adsorbent increases removal efficiency whereas removal efficiency of MB decreases with increasing MB concentration. Hence, it can be understood that the effect of adsorbent dosage is dominant compared to the initial MB concentration. Moreover, it can be observed from the 3D pictorial representation that the maximum removal efficiency of 99.9% is projected as adsorbent dosage and MB concentration interact. However, the minimum removal efficiency projected of 89.4% was found.

#### Adsorbent dosage and contact time

**Figure 7:**
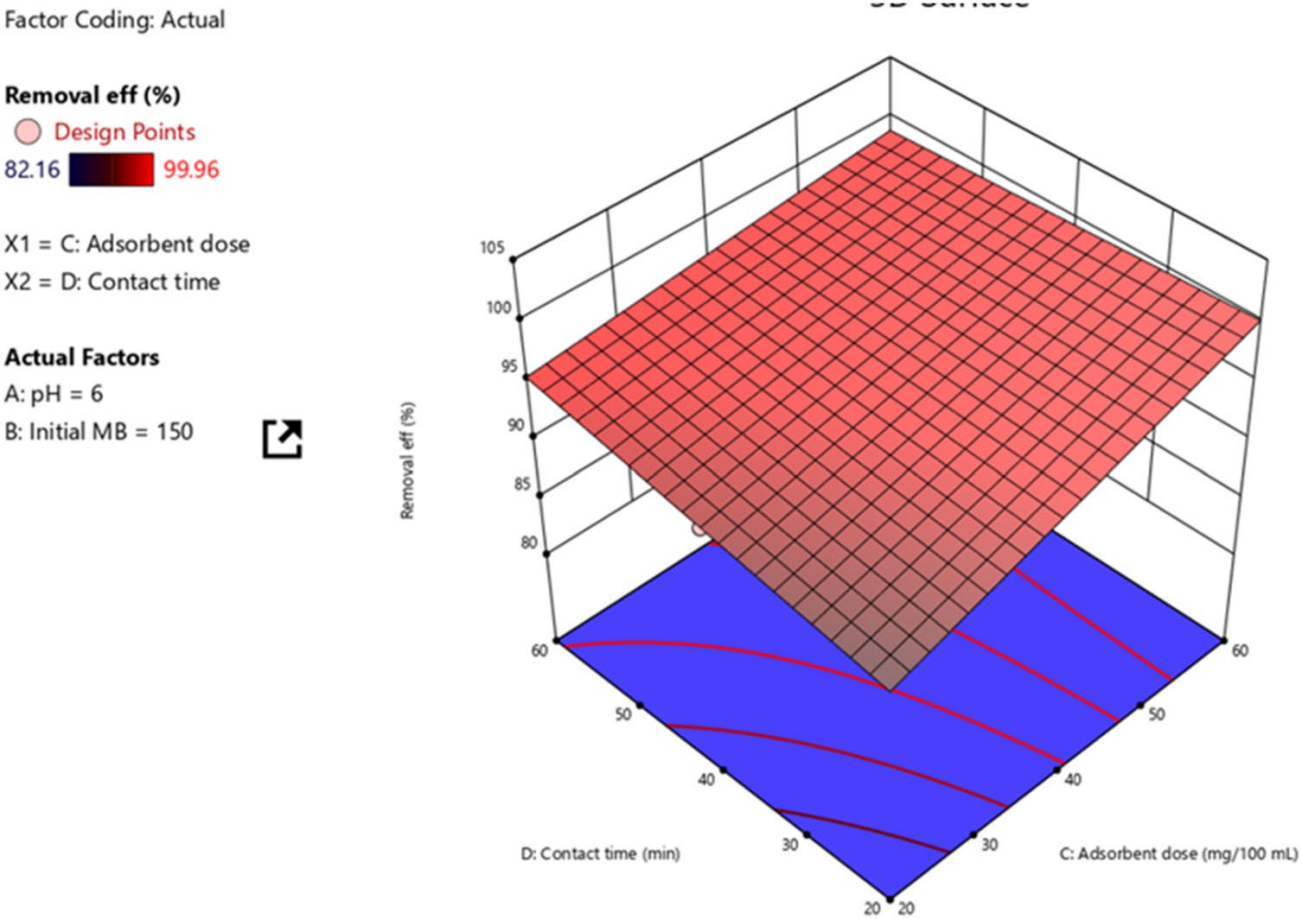
Interaction effect of adsorbent dosage and contact time.

The interaction effects of adsorbent dosage and contact time were evaluated using the response surface methodology. This was conducted by setting the pH and initial MB concentrations at middle values while varying the initial MB concentration and contact time as shown in Fig. 7. The 3D pictorial representation of the interaction effect of the adsorbent dosage and contact time illustrated the negative effect. Individually increasing both adsorbent dosage and contact time increased the removal efficiency of MB dye. Moreover, the projected removal efficiency ranged from 90.7 to 98.9%. The maximum projected removal efficiency of 98.9% was attained at the treatment points of adsorbent dosage of 60 mg/100 mL and 60 min contact time. Hence, the projected value is in line with the experimentally determined values. These show the adjusted and actual values are almost equal.

#### pH and contact time

**Figure 8:**
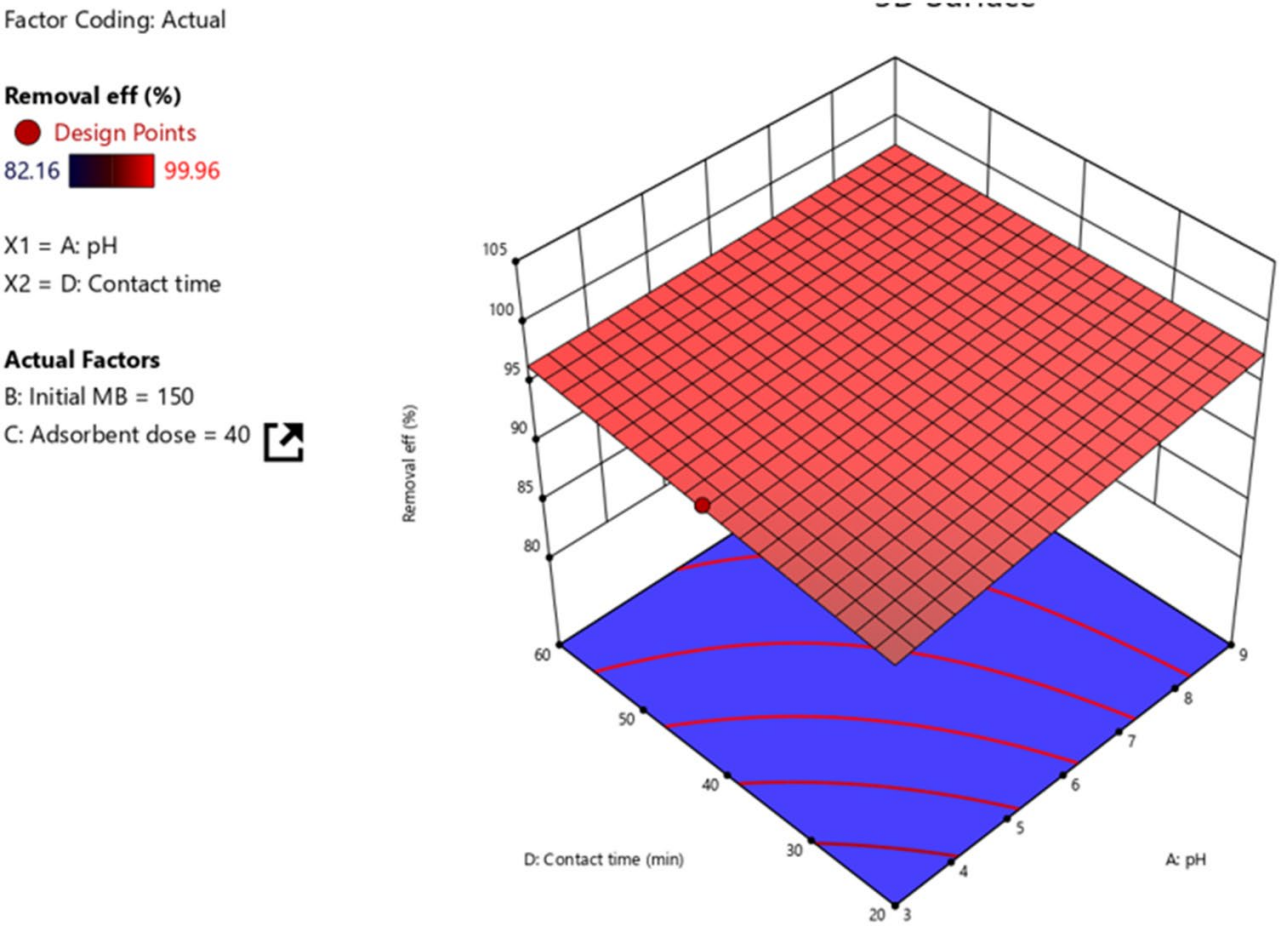
Interaction effect of contact time and pH.

pH and contact time are among the various factors influencing the adsorption performance. Normally, increasing both pH and contact time increases the adsorption of cationic dyes like MB. As contact time increases, there will be sufficient time to adsorb MB onto *Rumex* *abysiniccus*-derived activated carbon thereby increasing the adsorption efficiency. However, after a certain contact time, there is a point where an increase in contact time will not bring a significant change in removal efficiency. As indicated in the 3D pictorial representation depicts in Fig. 8. The adsorption of MB onto *Rumex* *Abyssinicus* was favoured by the interaction of pH and contact time indicating the interaction effect positive.

#### pH and adsorbent dosage

**Figure 9:**
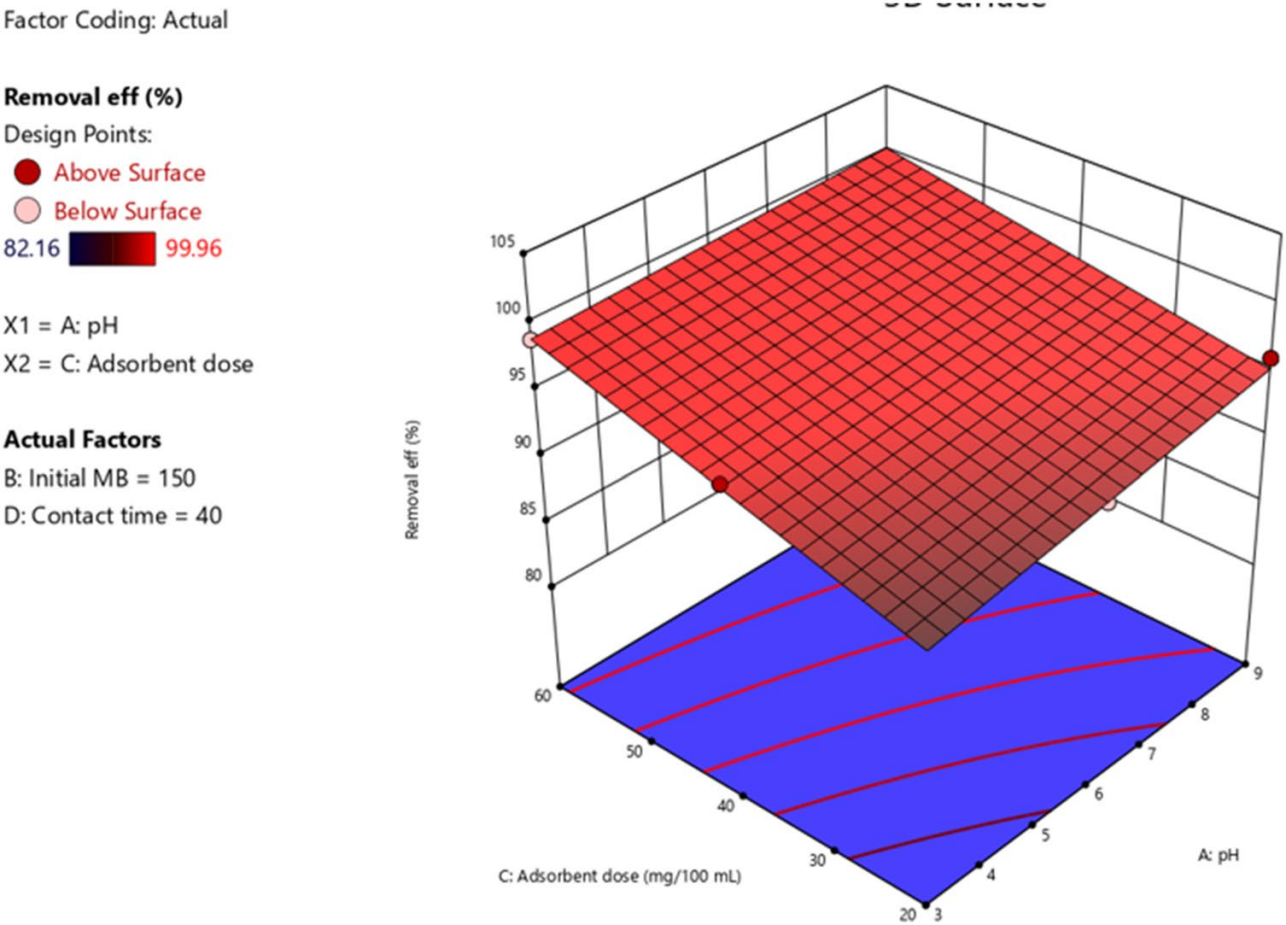
Interaction effect of adsorbent dosage and pH.

To effectively design the adsorption plant, the pH and adsorbent dosage of the adsorption process should be carefully considered. This is because to be economical, the minimum amount of adsorbent dose has to be utilized to bring significant adsorption capacity. The 3D pictorial representation of the effect of pH and adsorbent dosage shown in Fig. 9 revealed the interaction of pH and adsorbent dosage. This interaction was a bit different and the negative impact of the adsorption of MB onto activated carbon of *Rumex* *Abyssinicus* was observed*.* This interaction practically suppressed the adsorption performance.

### Adsorption isotherm

**Figure 10:**
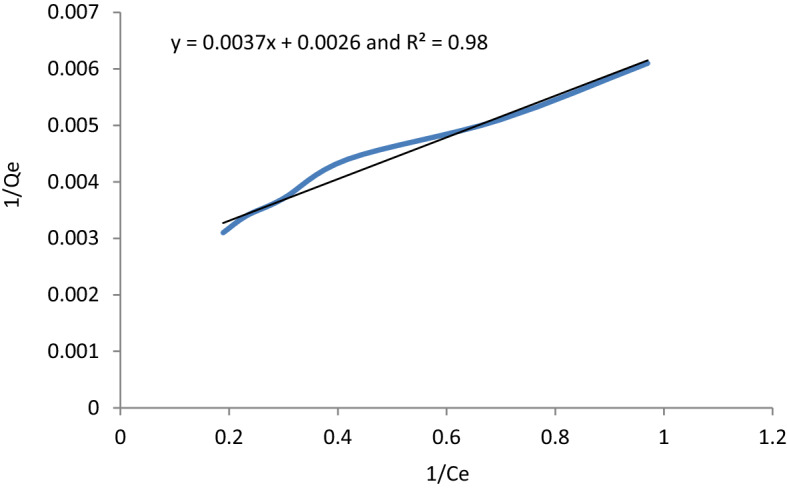
Langmuir isotherm graphical plot for MB removal.

**Figure 11:**
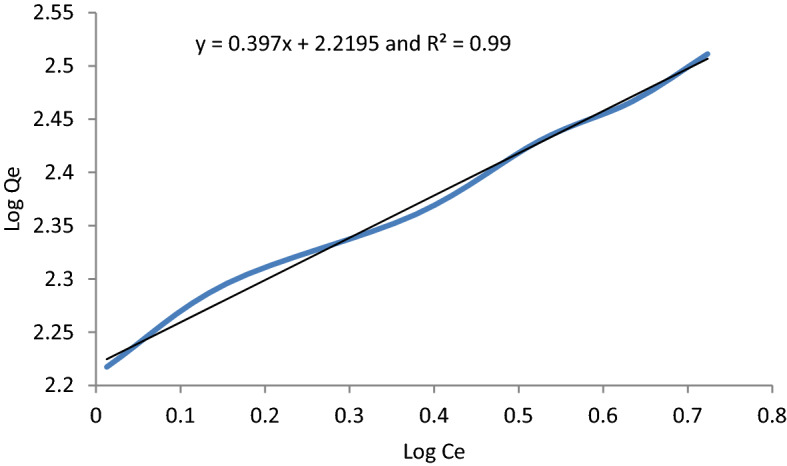
Freundlich isotherm graphical representation.

**Figure 12:**
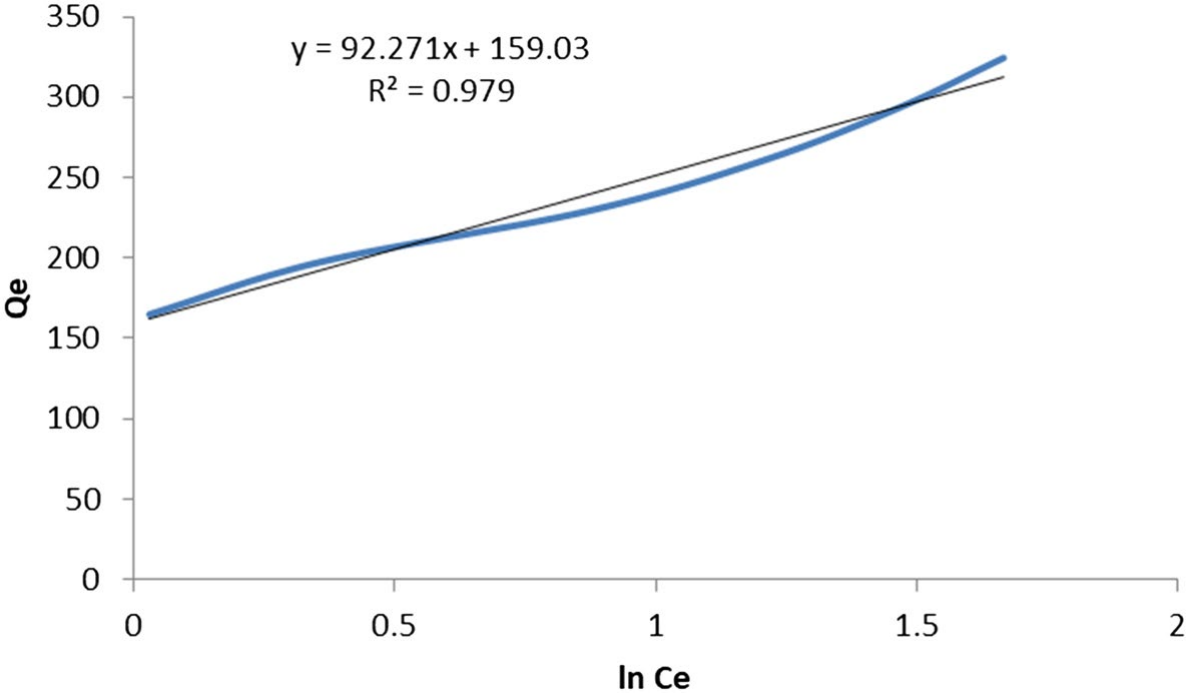
Temkin isotherm model for MB removal onto *Rumex* *abyssinicus.*

The graphical representation of the Langmuir, Freundlich, and Temkin isotherms was presented in Figs. 10, 11, and 12 respectively. The coefficients of determinant R^2^ for Langmuir, Freundlich and Temkin isotherms are 0.982, 0.9935, and 0.979, respectively. The data best fitted the Freundlich isotherm, showing the adsorption process as multilayer and heterogeneous. Hence, the surface of the adsorbent is heterogeneous, and the adsorption process of MB occurs on the active site of the multilayer surface of the adsorbent. According to Freundlich isotherm, as methylene blue binds to the active site of adsorbent, adsorption energy exponentially decreases. Additionally, the adsorption space accommodates more than one layer. This shows that most of the dye molecules are in direct contact with the adsorbent materials while some of them are attached to pre-adsorbed material. Moreover, the Qmax and KL of the Langmuir isotherm are 387.56 mg/g and 0.40, respectively. The Qmax value recorded in this study was high showing the effectiveness of the adsorption process. The K_L_ value of the Langmuir type isotherm model not only signifies adsorption capacity but also expresses the affinity of adsorbent toward adsorbate. In this regard, strong binding is indicated by high values of K_L_. Additionally, a higher amount of K_L_ value implies less free energy requirement. In this study, the K_L_ value of 0.4 mg/g is indicative of weak binding of adsorbate and adsorbent which in turn gives a clue that the physical adsorption mechanism and reversibility of the adsorption process. On the other hand, the Langmuir isothermal feasibility (RL) and Freundlich isotherm constant related to intensity (1/n) were determined to be 0.66 and 0.397, suggesting that the adsorption process is favorable. In this study, the 1/n value recorded was 0.397, which is low, inferring higher heterogeneity. In favorable Freundlich isotherm, i.e. n > 1, active sites with the highest binding energies would be used first for less heterogeneous surfaces, and then pursued by weaker sites for more heterogonous surfaces. The present study resulted in a higher Freundlich adsorption capacity KF of 165.77 mg/g. Besides, a higher K_f_ value shows less free energy requirement for the adsorption process. The Temkin isotherm constants AT and BT are determined to be 5.669 L/g and 26.883 J/mol, respectively. The heat of sorption calculated from the Temkin isotherm was determined to be 0.006425 kcal/mol, which is less than 1, indicating physical adsorption. In line with this, the Freundlich model goodness-of-fit is supported by the regression coefficient, with an R^2^ value of 0.96. Generally, the Adsorption isotherm is intended to understand the adsorption mechanism which describes the distribution of adsorbed molecules on the adsorbent interface. The prediction of the adsorption mechanism is not straightforward and is also highly influenced by the nature of the pollutants such as dissociated ions, neutral molecules, polar, non-polar, hydrophobicity, and hydrophilicity. However, the adsorption mechanisms can be described by the hydrophobic effect, π–π electron donor–acceptor, covalent bonding, coulombic interaction, H-bonding, π-interaction, surface complexation, electrostatic interactions, ion exchange, dipole interactions. These can be demonstrated by coordination formation, the dis/appearance or shifting of the functional (crystal peaks), and the occurrence of the surface precipitate. The adsorption MB was a heterogeneous, multilayer, and physical process which could be demonstrated by van der Waals forces, hydrogen bonding, and hydrophobic interactions.

### Adsorption kinetics

Kinetics studies are conducted to determine how fast the adsorption process takes place. Moreover, it is also useful in evaluating whether the data best fits a pseudo-first-order, pseudo-second-order, or Intraparticle diffusion model. This is used to evaluate the rate-determining step in surface adsorption where physicochemical interaction between adsorbate and adsorbent takes place. In the current study, the adsorption kinetics data were fitted with a pseudo-first-order, a pseudo-second-order, and a diffusion model. According to the determinant coefficient R^2^, Q_e_ experimental and Q_e_ calculated values obtained from the linear fit of these models. Then, pseudo-second-order kinetics was found to be more explanatory with an R^2^ of 0.88 among the three kinetic models. This indicates the adsorption rate is more dependent on the adsorption capacity compared to the adsorbate concentration. As indicated in Table 5, the slope of the intraparticle diffusion model was not 0.5 indicating that this model not exclusively controls the rate of reaction nevertheless it has participated in the adsorption process. In pseudo-second-order kinetics, the adsorption rate is more dependent on adsorption capacity not on the concentration of adsorbate. This phenomenon is in good agreement with the effects adsorption variables observed. Similarly, in this study, the effect of adsorbent dosage was superior compared to adsorbate concentration. Hence, extra granular diffusion may control the adsorption process. Fundamentally, the transfer of a solute from the solution to the adsorbent material can be governed by four main kinetic mechanisms. The first two mechanisms include the bulk movement of adsorbate molecules and film diffusion. Normally, bulk diffusion is faster compared to film diffusion which is why this mechanism is not considered in designing adsorption kinetic systems. The movement of adsorbate from the surface of the adsorbent to the porous adsorbent and rapid adsorptive attachment of adsorbate on the pores active site of adsorbent are the last two adsorption mechanisms. Since the adsorption system of MB onto Rumex abyssiniccus derived, activated carbon was found to have characteristics of small adsorbate size, poor mixing, and film diffusion which can be taken as rate controlling step.

**Table 5:** Kinetics parameters for adsorption of MB onto *Rumex* *abysiniccus* activated carbon.

Kinetic model	Kinetic parameters	Kinetic parameter values
Pseudo first order	R^2^	0.83
	K_1_	0.091
	Qe calculated	150.31
	Equation	Y = − 0.0398X + 2.177
Pseudo second order	R^2^	0.88
	K_2_	−0.00128
	Qe calculated	149.25
	Equation	Y = 0.0067X − 0.035
Intraparticle diffusion model	R^2^	0.94
	K_p_	3.162278 ${\mathrm{e}}^{-116}$
	Intercept	115.5
	Equation	Y = 6.5683X + 115.5

## Conclusions

*Rumex* *abyssinicus* activated carbon was successfully prepared by chemical activation followed by thermal activation methods. This adsorbent-activated carbon showed excellent chrematistics of a good adsorbent such as high surface area, conducive surface morphology, high fixed carbon, and multifunctionalities. The response surface methodology coupled with the Box-Behnken approach was used to optimize the adsorption process. The removal efficiency of MB ranged from 82.2 to 99.9%. The maximum removal efficiency of 99.9% was attained at optimum conditions of pH 9, contact time of 60 min, an initial MB concentration of 100 mg/L, and an adsorbent dosage of 60 mg/100 mL. Moreover, the maximum adsorption capacity of 322 mg/g recorded in this study shows that a small amount of the adsorbent was used to achieve significant removal of MB from an aqueous environment. It was also found that the Freundlich isotherm model and pseudo-second-order kinetics best explain the adsorption of MB onto the activated carbon of *Rumex* *abyssinicus*. This shows that the adsorbent surface is heterogeneous and multi-layered, and the nature of the adsorption is physical. In general, this adsorbent material is promising to be scaled up at the industrial level. However, further investigation such as adsorbent development optimization, column analysis, adsorbent composite with magnetic material, and thermodynamics study should be carried out before pilot scale test and industrial application for wastewater treatment.

## Data Availability

All data are fully available without restriction and can be obtained from the corresponding author.
